# Stimuli‐Responsive Nanotechnology for RNA Delivery

**DOI:** 10.1002/advs.202303597

**Published:** 2023-11-01

**Authors:** Hui Zhou, Dean Shuailin Chen, Caleb J. Hu, Xuechuan Hong, Jinjun Shi, Yuling Xiao

**Affiliations:** ^1^ Department of Cardiology, Clinical Trial Center Zhongnan Hospital of Wuhan University School of Pharmaceutical Sciences Wuhan University 430071 Wuhan China; ^2^ Center for Nanomedicine and Department of Anesthesiology Perioperative and Pain Medicine Brigham and Women's Hospital Harvard Medical School Boston MA 02115 USA; ^3^ State Key Laboratory of Organic Electronics and Information Displays & Institute of Advanced Materials (IAM) Nanjing University of Posts & Telecommunications 210023 Nanjing China

**Keywords:** gene silencing, genome editing, nanotechnology, protein expression, RNA delivery, stimuli‐responsive

## Abstract

Ribonucleic acid (RNA) drugs have shown promising therapeutic effects for various diseases in clinical and preclinical studies, owing to their capability to regulate the expression of genes of interest or control protein synthesis. Different strategies, such as chemical modification, ligand conjugation, and nanotechnology, have contributed to the successful clinical translation of RNA medicine, including small interfering RNA (siRNA) for gene silencing and messenger RNA (mRNA) for vaccine development. Among these, nanotechnology can protect RNAs from enzymatic degradation, increase cellular uptake and cytosolic transportation, prolong systemic circulation, and improve tissue/cell targeting. Here, a focused overview of stimuli‐responsive nanotechnologies for RNA delivery, which have shown unique benefits in promoting RNA bioactivity and cell/organ selectivity, is provided. Many tissue/cell‐specific microenvironmental features, such as pH, enzyme, hypoxia, and redox, are utilized in designing internal stimuli‐responsive RNA nanoparticles (NPs). In addition, external stimuli, such as light, magnetic field, and ultrasound, have also been used for controlling RNA release and transportation. This review summarizes a wide range of stimuli‐responsive NP systems for RNA delivery, which may facilitate the development of next‐generation RNA medicines.

## Graphical abstract

Ribonucleic acid (RNA) therapeutics have demonstrated great potential for treating various diseases. Nanoparticle delivery technologies can protect RNAs from degradation, increase cellular uptake and cytosolic transportation, prolong systemic circulation, and improve tissue/cell targeting. The review summarizes a wide range of stimuli‐responsive nanoparticle systems for RNA delivery, highlighting their unique features and offering insights into the development of next‐generation RNA nanomedicines.

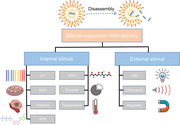

## 1 Introduction

Traditional small‐molecule drugs and antibody drugs primarily bind to target proteins, but the “undruggable” properties of many proteins limit the development of these two types of therapies. According to a previous report, ∼3000 of the ∼20 000 proteins encoded in the human genome are druggable, and to date, only ∼700 corresponding drugs based on druggable proteins have been developed.^[^ ^1^ ^]^ Moreover, antibody drugs are generally limited to targeting cell membrane proteins or extracellular proteins. Ribonucleic acids (RNAs) have been proposed as a promising alternative class of therapeutic molecules to overcome these limitations because they can regulate gene and/or protein expression within target cells and could thus be used to treat various diseases such as genetic disorders, infectious diseases, cardiovascular diseases, metabolic diseases, and cancers. Several small RNAs, such as antisense oligonucleotide (ASO), small interfering RNA (siRNA), and microRNA (miRNA), can induce gene silencing based on the principle of complementary base pairing. With proper sequence design, these small RNAs could in principle target any gene of interest. Messenger RNA (mRNA), which has a much larger molecular weight than short non‐coding RNAs, carries the genetic information necessary for protein synthesis. There are many other types of RNAs with their own unique functions.

Despite their tremendous potential for biomedical applications and disease treatment, the delivery of RNA drugs is hindered by various cell and tissue barriers, thus limiting their efficacy.^[^ ^2^, ^3^ ^]^ For example, naked RNA drugs are sensitive to endogenous nucleases in blood and tissues and can be rapidly filtered by renal clearance, leading to a short half‐life. Also, RNAs are negatively charged macromolecules, which may not be able to readily cross cellular membranes.^[^ ^4^, ^5^ ^]^ In addition, after endocytosis of RNAs, only those that escape from the endosomes can exert their biological effects.^[^ ^6^ ^]^ Therefore, various approaches have been developed to improve RNAs’ stability and delivery. Modifications of the sugar unit, the nucleobase, nucleic acid terminals, and linkage are common methods to enhance the biostability of RNAs and to improve their in vivo properties.^[^ ^7^ ^]^ For example, some common sugar modifications employed in RNA therapeutics involve substituting the endogenous 2′‐hydroxy group with a 2′‐fluoro, 2′‐O‐methyl, or 2′‐deoxy group. Common linker modifications replace the endogenous phosphodiester bonds with phosphorothioate or amide‐based linkages to enhance RNA resistance to nucleases. Based on these modification strategies, several ASO drugs have been clinically approved.^[^ ^8^ ^]^ Furthermore, the recently approved siRNA therapeutics and mRNA vaccines also contain chemical modifications.^[^ ^9^, ^10^ ^]^ In addition to chemical modification, another effective strategy to improve RNA delivery is through conjugation with targeting ligands. For instance, various types of ligands such as vitamin E,^[^ ^11^ ^]^ N‐Acetylgalactosamine (GalNAc),^[^ ^12^ ^]^ aptamer,^[^ ^13^ ^]^ antibody,^[^ ^14^ ^]^ and cell‐penetrating peptide^[^ ^15^ ^]^ have been attached to RNAs to improve their delivery and therapeutic efficacy. Among these ligands, GalNAc (an amino sugar derivative of galactose) remains the most successful example of short RNA conjugates, with several siRNA‐GalNAc conjugates recently approved for clinical use.^[^ ^16^ ^]^

In parallel to chemical modification and conjugation strategies, nanoparticle (NP) technology has also been widely explored for RNA delivery and has contributed to the clinical success of the first siRNA therapy (Onpattro) and two mRNA vaccines (mRNA‐1273 by Moderna and BNT162b2 by Pfizer/BioNTech). NP delivery vehicles mainly include liposomes, lipid NPs (LNPs), micelles, polymeric NPs, dendrimers, peptides, and inorganic NPs.^[^ ^17^, ^18^, ^19^, ^20^ ^]^ While each of these NP types has its own unique properties and applications, many of them are capable of protecting RNAs from nuclease degradation, increasing cellular uptake and endosomal escape, and/or prolonging systemic circulation. For example, lipid‐based NPs, including liposomes and LNPs, have demonstrated many clinical successes in drug delivery, with LNPs now approved for siRNA delivery for gene silencing and mRNA delivery for vaccination.^[^ ^21^, ^22^ ^]^ Polymeric NPs also provide a versatile delivery platform for RNAs, by robustly tailoring the polymer composition and hydrophobicity/hydrophilicity.^[^ ^23^, ^24^ ^]^ Exosomes and other naturally occurring vesicles are being increasingly explored for their potential as RNA carriers, leveraging their biocompatibility and intrinsic ability to interact with specific cell types.^[^ ^25^, ^26^ ^]^ All these collectively contribute to the diversity of delivery options for the clinical development of RNA therapy.

Stimuli‐responsive nanotechnology has also received considerable interest for systemic and local delivery of various RNAs, owing to its unique features like controlled/triggered release of cargos to improve transfection efficiency and tissue/cell specificity. Specific internal stimuli in the tissue microenvironment of cells include low pH, increased redox potential, overexpression of specific enzymes, hypoxia, high adenosine triphosphate (ATP) level, and temperature change.^[^ ^27^ ^]^ External stimuli such as light irradiation, ultrasound, and magnetic fields can also be applied to the desired site. When exposed to these stimuli, the responsive NPs could undergo structural or conformational changes, protonation, or cleavage, thus enhancing RNA delivery. Moreover, stimuli‐responsive nanoplatforms could improve tissue targeting, promote endosomal/lysosomal escape, and increase the intracellular release efficiency of RNA drugs, thus improving treatment efficacy and reducing toxicity and side effects. Despite the significant efforts and interests in this unique area, no comprehensive review has been put forward. Herein, we provide an overview and discussion of various stimuli‐responsive strategies based on both internal stimuli [e.g., pH, glutathione (GSH), reactive oxygen species (ROS), enzyme, ATP, hypoxia, and heat] and external stimuli (e.g., light, ultrasound, and magnetic), aiming to provide guidance and insight into the development of safer and more efficient NP delivery systems for RNA therapeutics.

## 2 Classification of RNA Therapeutics

**Figure 1:**
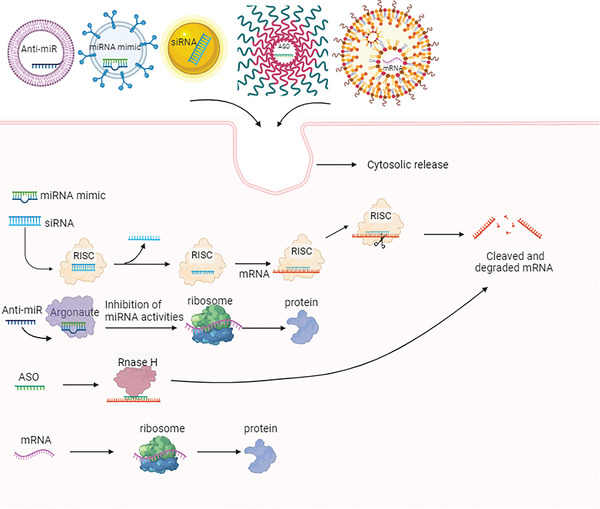
Schematic of general mechanisms of ASO, siRNA, miRNA mimic, anti‐miR, and mRNA for modulating protein expression. This figure was created with BioRender.com.

Several types of RNA therapeutics are clinically approved or have moved to clinical trials, such as ASO, aptamers, siRNA, miRNA, mRNA, and self‐amplifying RNA(saRNA).^[^ ^28^ ^]^ In addition, new types of RNA therapeutics including circular RNA (circRNA) and transfer RNA (tRNA) have also recently received considerable attention. Below, we briefly summarize the major types of RNA therapeutics (**Figure** **1**).

### 2.1 ASO

ASOs are short single‐stranded nucleic acids (DNA, RNA, or mixtures), which are complementary to a certain region of RNA, and thus can regulate mRNA expression by RNase H‐mediated pre‐mRNA cleavage and/or steric hindrance of ribosomes to suppress translation.^[^ ^29^ ^]^ In addition, ASOs can also induce exon‐skipping by modulating pre‐mRNA splicing. Since the approval of the first ASO drug (Fomivirsen) for cytomegalovirus retinitis in immunocompromised AIDS patients,^[^ ^30^ ^]^ ∼10 are now in clinical use and many are in late‐stage trials.

### 2.2 siRNA

siRNA is a double‐stranded RNA of ∼20–25 nucleotides in length. Gene silencing induced by siRNA‐mediated RNA interference (RNAi) is an essential method of gene expression regulation. Its mechanism consists of the cleavage of exogenous double‐stranded RNA into siRNA by the Dicer enzyme. siRNA (or directly introduced synthetic siRNA) then binds to Argonaute proteins in the cytoplasm to form RNA‐induced silencing complex (RISC), and then the siRNA is unwound, the sense chain is cleaved and degraded, while the RISC binding antisense chain is activated to specifically bind target mRNA. This results in specific degradation and cleavage of the target mRNA, blocking the translation of specific genes and inhibiting gene expression.^[^ ^31^ ^]^ Since RNAi was discovered in the 1990s, siRNAs have become a ubiquitous and compelling tool to silence the expression of virtually any gene in a highly efficient and specific manner, including those traditionally considered undruggable targets. The first siRNA drug (Patisiran) was approved by the U.S. Food and Drug Administration (FDA) in 2018.

### 2.3 miRNA

miRNA is an endogenous non‐coding single‐stranded small‐molecule RNA with a length of 18–25 nucleotides. miRNA are abundant in plants, animals, human body fluids, and some viruses, and may act as biomarkers for various diseases. miRNAs function via base‐pairing, with complementary sequences found inside mRNA molecules.^[^ ^32^ ^]^ As a result, these mRNA molecules are silenced by one or more of the following processes: (1) cleavage of the mRNA strand into two pieces, (2) destabilization of the mRNA through shortening of its poly(A) tail, and (3) less efficient translation of the mRNA into proteins by ribosomes.^[^ ^33^, ^34^ ^]^ Furthermore, miRNA‐based strategies, in the form of miRNA inhibitors (also anti‐miR) and miRNA mimics, can regulate both the expression and the repression of multiple genes. Synthetic double‐stranded miRNA mimics are similar to siRNAs of the RNAi pathway, suppressing the expression of mRNAs. In contrast, single‐stranded anti‐miRs can sequence‐specifically bind to mature miRNAs to interrupt their function on mRNAs.^[^ ^17^ ^]^ With the advances in miRNA biology and the development of delivery nanotechnology, miRNA‐based therapeutics have been broadly explored in preclinical research, and some have reached clinical development.

### 2.4 mRNA

An mRNA molecule is a single‐stranded RNA complementary to the genetic sequence and may be read by a ribosome during the protein production process. mRNA is produced during the transcription process when an enzyme (RNA polymerase) converts a gene to the primary transcript mRNA (also known as pre‐mRNA). In most cases, this pre‐mRNA still includes introns or segments that do not contain the final amino acid sequence. These are spliced out during the RNA splicing process, leaving just the exons, the protein‐coding portions. mRNA also has many applications due to its function. For example, a cell may create a protein employed directly to treat or prevent sickness by delivering a nucleoside‐modified mRNA sequence into a cell. More indirectly, the protein can stimulate the development of an endogenous stem cell in the desired direction.^[^ ^35^ ^]^ In addition to the clinical success of mRNA vaccines for COVID‐19, mRNA could also be used in many other diseases such as cancer. For example, mRNA NPs have recently been used to restore tumor suppressors, such as PTEN^[^ ^36^, ^37^ ^]^ and p53,^[^ ^38^, ^39^ ^]^ for cancer treatment. Most mRNA drug candidates are currently in early‐stage clinical trials. Furthermore, a number of mRNA vaccines, either alone or in combination with drugs for the treatment of various types of cancer, including pancreatic cancer, colorectal cancer, and melanoma, are also being tested in clinical trials.

### 2.5 Other RNAs

In addition to the RNAs mentioned above, there are some other RNAs with therapeutic potential. For example, aptamers are short single‐stranded DNA or RNA molecules that selectively bind to a specific target, including proteins, peptides, carbohydrates, and small molecules; they are widely used in medicine and biotechnology.^[^ ^40^ ^]^ Aptamers exhibit significant advantages in size, synthetic accessibility, and modification by medicinal chemistry. Various aptamers are currently in development and may change how nucleic acid therapeutics are perceived. It is promising that aptamers will be used more with other therapeutic molecules and modalities in the future. Circular RNA (circRNA), a type of non‐coding single‐stranded RNA, is covalently closed to form a continuous loop.^[^ ^41^ ^]^ It has been shown that circRNAs are abundant and evolutionarily conserved, and many of them exert important biological functions by acting as transcriptional regulators or, miRNA/protein inhibitors (“sponges”), by regulating protein function, or by being translated themselves.^[^ ^41^, ^42^ ^]^ In addition to their crucial roles in various diseases, making them appealing diagnostic biomarkers and therapeutic targets,^[^ ^43^, ^44^ ^]^ circRNA has also been explored as an alternative to mRNA for protein generation.^[^ ^45^, ^46^, ^47^, ^48^ ^]^ It shows higher stability than linear mRNA because of its closed‐loop structure. Transfer RNA (tRNA) is a small RNA molecule (typically 76–90 nucleotides in length) that plays an important role in protein synthesis. tRNAs function at specific sites in the ribosome during translation, which is a process that synthesizes a protein from an mRNA molecule. Dysregulation of tRNAs, mediated by alterations in their abundance or function, is associated with several distinct human diseases,^[^ ^49^, ^50^ ^]^ including neurological disorders and cancer. Therefore, it is important to maintain precise control of functional tRNA levels. Recently it has been revealed that suppressor tRNAs hold the potential for treating or preventing hereditary cancer syndromes caused by nonsense mutations^[^ ^49^ ^]^ and other diseases,^[^ ^50^, ^51^ ^]^ leading to the clinical development of suppressor tRNA therapies by several biotechnology companies.

## 3 Internal Stimuli‐Responsive Systems for RNA Delivery

Materials science has revolutionized the central paradigm of drug delivery, such that the physicochemical properties of delivery systems could be customized to develop “smart” or “intelligent” systems that can deliver the therapeutic molecule on demand. In addition, a growing understanding of abnormal tissue microenvironments and the exploitation of subtle differences in the biological milieu has inspired advances in designing materials for drug delivery.

Traditional RNA drug delivery systems rely heavily on cationic/ionizable materials to load RNA drugs to form nanocomposites and improve cellular uptake and endosomal escape.^[^ ^52^ ^]^ In order to improve stability and blood circulation, polyethylene glycol (PEG) is often used to shield or neutralize the surface charge of the carrier and reduce opsonin absorption.^[^ ^53^ ^]^ However, when this type of carrier reaches the target tissue, PEG may reduce the cellular uptake of the drug carrier and endosomal escape.^[^ ^54^ ^]^ In addition, the insufficient release of RNA drugs in target cells such as tumor cells by delivery NPs may also reduce the treatment efficacy. Therefore, a microenvironment‐responsive carrier with stable loading and high‐efficiency responsive drug release characteristics may be key to addressing the problem of RNA drug delivery in vivo.

To overcome the biological barriers, many smart formulations have been developed in which stimuli‐responsive moieties translate chemical or internal physical signals into significant behavior changes, such as swelling, aggregation, degradation, surface rearrangement, and charge reversal. Remarkable variations in physiological parameters exist at the organ, tissue, and cell levels, and are highly correlated with various pathological conditions, which can serve as attractive biomarkers for diagnosis and natural endogenous stimuli for controlled drug release in cancer, infection, diabetes, as well as cardiovascular and degenerative diseases. Among these nanocarriers, common response strategies include: 1) in response to overexpression of specific enzymes or pH environment outside abnormal cells, increasing cell uptake of NPs or through size’ transformation using “PEG shedding” and charge reversal, and increasing the ability of NPs to penetrate and accumulate in specific targeted tissues; 2) in response to the acid environment in endosomes/lysosomes, enhancing the endosome's escape ability of NPs through the protonation of ionizable groups; 3) in response to the hypoxic environment or overexpression of factors such as GSH, ROS, and ATP in target cells, accelerating dissolution of NPs to improve the release efficiency of RNA drugs in the cytoplasm. Herein, the design strategies of various internal stimuli‐responsive RNA drug delivery systems are discussed (**Table** **1**).

**Table 1:** Internal stimuli‐responsive RNA drug delivery systems.

Responsive mode	Responsive group	Chemical structure	RNA type^[reference]^
pH‐responsive	Maleic acid amide	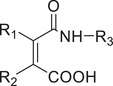	siRNA^[^ ^55^, ^56^ ^]^
	Ketal, Acetal	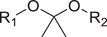	siRNA,^[^ ^57^, ^58^ ^]^ mRNA^[^ ^59^ ^]^
	Imine	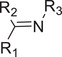	siRNA^[^ ^60^ ^]^
	Tertiary amine	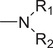	siRNA^[^ ^61^, ^62^, ^63^, ^64^ ^]^
	Poly(β‐amino ester)	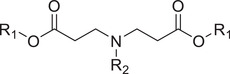	siRNA,^[^ ^65^, ^66^ ^]^ mRNA^[^ ^67^ ^]^
ROS responsive	Thioketal	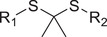	siRNA^[^ ^68^, ^69^, ^70^, ^71^ ^]^
	Boronic esters	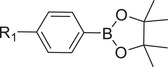	siRNA,^[^ ^72^, ^73^ ^]^ miRNA^[^ ^74^ ^]^
	Diselenide	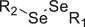	siRNA^[^ ^75^, ^76^ ^]^
GSH responsive	Disulfide	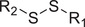	siRNA,^[^ ^77^, ^78^, ^79^ ^]^ miRNA,^[^ ^80^ ^]^ mRNA^[^ ^81^ ^]^
	Diselenide	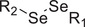	siRNA^[^ ^82^ ^]^
Hypoxia responsive	Nitroimidazole	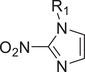	siRNA^[^ ^83^, ^84^ ^]^
	Azobenzene	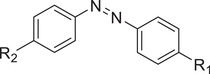	siRNA^[^ ^85^, ^86^ ^]^
Temperature responsive	N‐isopropylacrylamide	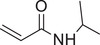	siRNA^[^ ^87^, ^88^, ^89^ ^]^
Enzyme responsive	Peptide (MMP‐2 responsive)	GPLGAIAGQ	siRNA^[^ ^90^ ^]^, miRNA^[^ ^91^ ^]^
	Peptide (MMP‐7 responsive)	VPLSLYSGCG	siRNA^[^ ^92^ ^]^
	Poly(α)glutamate (cathepsin responsive)	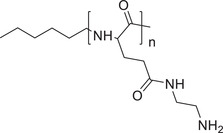	siRNA^[^ ^93^ ^]^
ATP responsive	Phenylboric acid	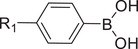	siRNA,^[^ ^94^, ^95^ ^]^ mRNA^[^ ^96^ ^]^

GSH: glutathione; MMP: Matrix metalloproteinase; ROS: Reactive oxygen species; ATP: Adenosine triphosphate

### 3.1 Glutathione Tripeptide‐Responsive Nanocarriers

Tumors exhibit characteristic oxidative extracellular and reductive intracellular environments, generating redox potentials, which are the driving force for the development of redox‐response delivery materials and vehicles. Redox‐responsive systems tend to lose their structural integrity in response to the remarkably higher cytosolic and nucleus concentration of glutathione tripeptide (GSH) (∼1–10 mm) compared to the extracellular matrix (∼2–20 µm).^[^ ^97^ ^]^ Intracellular GSH maintains its reduced state under the action of NADPH and glutathione reductase, which is the main intracellular antioxidant and plays an important role in normal physiological activities. Tumor cells exhibit higher concentrations of GSH, at least four times that of normal cells.^[^ ^98^ ^]^ Therefore, using GSH‐responsive materials to construct nano‐systems could improve the intracellular release of RNA drugs and enhance their efficiency based on the significant difference in GSH concentration inside and outside tumor cells.^[^ ^99^ ^]^

#### 3.1.1 Disulfide Cross‐Linked Polyplexes

**Figure 2:**
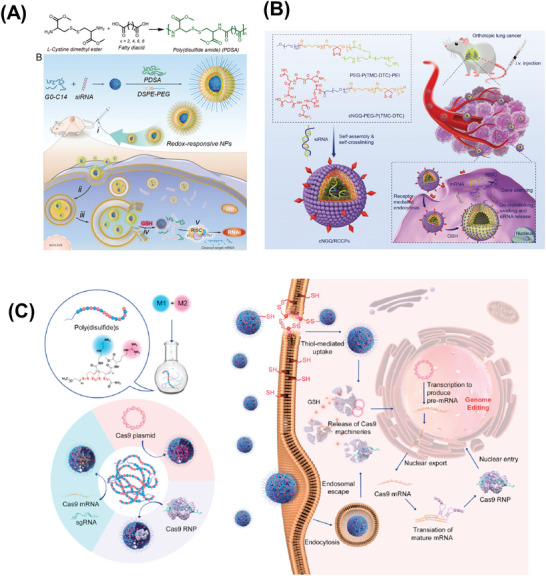
A) Synthesis scheme of the PDSA polymer and the PDSA NPs for systemic siRNA delivery. Reproduced with permission.^[^ ^78^ ^]^ Copyright 2018, John Wiley and Sons. B) Illustration of GSH‐responsive polymers for siRNA delivery to orthotopic lung tumors. Reproduced with permission.^[^ ^77^ ^]^ Copyright 2017, John Wiley and Sons. C) Preparation of poly(disulfide)s and their intracellular delivery processes for genome editing. Reproduced with permission.^[^ ^81^ ^]^ Copyright 2021, American Chemical Society.

Disulfide bonds (S─S) are the most broadly studied redox‐sensitive bonds, which can be used to prepare delivery systems like polymers, lipids, or proteins‐based systems.^[^ ^100^, ^101^ ^]^ Recently, the polymer poly(disulfide amide) (PDSA) composed of disulfide bonds was synthesized via the facile “one‐pot” method.^[^ ^78^ ^]^ The nanoplatform contained cationic lipid/PDSA and 1, 2‐distearoyl‐sn‐glycero‐3‐phosphoethanolamine‐poly(ethylene glycol) (DSPE‐PEG), which could encapsulate siRNA. This GSH‐responsive nanoplatform easily released siRNA in the cytoplasm because of disulfide bond cleavage (**Figure** **2**A). Also using the GSH responsiveness of disulfide bonds, amphiphilic polymers PEG‐b‐P(TMC‐DTC)‐PEI and cNGQ‐PEG‐b‐P(TMC‐DTC) were constructed to form nanocarriers to deliver siPLK1 in vivo for treating lung cancer. In the polymer mentioned above structure,^[^ ^77^ ^]^ PEI encapsulated siRNA in the nanocarrier through electrostatic interaction; the cyclic DTC groups can form disulfide bonds with each other. Peptide CNGQ, on the other hand, interacted with highly expressed α3β1 integrin on the surface of lung cancer cells to improve tumor cell targeting and siRNA uptake. When the nanocarrier enters the tumor cell, a high concentration of GSH in the cytoplasm will break the disulfide bond, resulting in the swelling of the nanocarrier and the rapid release of siRNA, which significantly improves the gene silencing efficiency of siRNA and the effect of inhibiting tumor growth (Figure 2B).

Several classes of reducible cationic polymers such as poly(disulfide amine)s, disulfide‐containing poly(amido amine), or histidine‐containing polycations based on disulfide bonds were also developed.^[^ ^102^, ^103^, ^104^ ^]^ Complexation of RNA with these polymers can significantly contribute to thiol‐mediated uptake and result in the efficient delivery of cargoes into the cytosol, which improved transfection or gene silencing compared with non‐redox sensitive analogs as a result of the quick disassembly of the complexes under reductive intracellular conditions. These kinds of PEG‐based polyplex micelles, which shed the surrounding PEG chains in response to a reducing environment, allow the full recovery of the cytosolic‐release properties of the polycation, thus leading to significant in vivo gene silencing. Other RNA‐delivery systems synthesized through disulfide linkages are siRNA‐grafted polymers^[^ ^105^ ^]^ and multimerized siRNA.^[^ ^106^ ^]^ Recently, a series of poly(disulfide)s through ring‐opening polymerization was synthesized for RNA delivery.^[^ ^81^ ^]^ This platform could envelop three forms of CRISPR‐Cas9 components (Cas9 plasmid, Cas9 mRNA, and sgRNA). It showed an excellent ability to deliver them, exhibiting efficient genome‐editing activities in vitro and in vivo^[^ ^81^ ^]^ (Figure 2C).

#### 3.1.2 Diselenide Cross‐Linked Polyplexes

**Figure 3:**
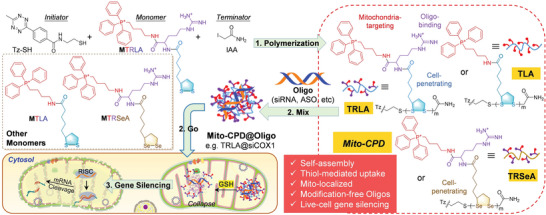
Diagram of Mito‐CPDs and their utilization in gene‐silencing. 1) Formation of Mito‐CPDs through a synthetic process; 2) Implementation of a “Mix‐and‐Go” technique to generate Mito‐CPD@Oligo complexes and facilitate their delivery into cells; 3) Suppression of gene expression within mitochondria. Reproduced with permission.^[^ ^82^ ^]^ Copyright 2023, John Wiley and Sons.

Because selenium (Se) belongs to the same family as sulfur (S) in the periodic table of elements, Se has also attracted extensive attention. Since selenium and sulfur have similarities in many respects, including electronegativity, atom size, and accessible oxidation states, diselenide bonds have been confirmed to be redox‐responsive like disulfide bonds. For example, three new monomers, MTLA, MTRLA, and MTRSeA, were designed by using disulfides or diselenides to form cleavable backbones of cell‐penetrating poly(disulfide)s (CPDs) for mtDNA gene‐silencing (**Figure** **3**). This nanoplatform (Mito‐CPD@Oligo) containing disulfide/diselenide‐bonded Mito‐CPDs was prone to depolymerization under reductive conditions, and showed successful mitochondrial delivery of siRNA and ASO for down‐regulation of target protein expression, leading to profound effects on mitochondrial functions.^[^ ^82^ ^]^ Diselenides may be more attractive given their higher redox sensitivity and better cell permeability than disulfides.

### 3.2 pH‐Responsive Nanocarriers

Tissues, organelles, and fluids in the human body have varying pH values. For instance, some tissues like the stomach, vagina, and tumor exhibit an acidic pH.^[^ ^107^ ^]^ In contrast, some other tissues/fluids like blood and tears have neutral or near‐neutral pHs. In addition, many disease pathologies are associated with pHs, which are different from those in healthy tissues.^[^ ^108^, ^109^, ^110^ ^]^ In addition to the weak acid environment of tumor tissues, intracellular organelles (e.g., endosomes and lysosomes) could also be acidic.^[^ ^111^, ^112^ ^]^ These differences in tissue/cell microenvironment provide the premise for regional drug delivery or site‐specific therapy with pH response to nanocarriers. Materials that respond to pH can usually undergo physical or chemical changes, such as swelling, contraction, dissociation, degradation, etc.^[^ ^107^ ^]^ The pH responsiveness comes from the protonation of ionizable groups or the acid degradation of chemical bonds. The introduction of pH‐triggered chemical bonds is one of the most widely used strategies for pH‐responsive nanodrug delivery systems. Commonly used pH‐sensitive bonds include phenylmethylimine bond, 2, 3‐dimethylmaleic anhydride, hydrazone bond, thiopropionate bond, imine bond, orthoester bond, ketal, acetal, and cycloacetal, etc.^[^ ^113^, ^114^, ^115^ ^]^ These bonds can be broken in acidic environments, allowing the carrier to degrade, thereby increasing uptake by tumor cells or speeding drug release.

#### 3.2.1 Acidic pH‐Responsive Protonatable Structures

Nucleic acids and their lipo‐/poly‐plexes are generally internalized by cells via endocytosis, followed by lysosomal digestion. Therefore, to circumvent the degradation of nucleic acids, lipo/‐poly‐plexes need to be designed to deliver them from the endosome to the cytosol. To this end, some lipids, polycations, or polymers have been developed to comprise amino groups with low pKa (5–7), enabling endosomal pH‐responsive protonation, and allowing RNA drugs to escape from the endosome, which is referred to as the proton sponge effect. Protonatable chemical groups include tertiary amines,^[^ ^116^ ^]^ imidazoles,^[^ ^79^ ^]^ and others. These chemical groups with different pKa values can capture or provide protons under specific pH conditions, resulting in pH‐dependent changes in these materials. When a pH‐dependent change occurs, it will increase membrane destabilization and promote endosomal escape of the NPs, leading to the disassembly of the carrier system and the release of RNA drugs.

pH‐mediated charge‐switchable lipids and polymers are widely used for RNA delivery, which has been well reviewed elsewhere.^[^ ^117^, ^118^, ^119^, ^120^ ^]^ For example, the pH‐responsive lipid 1,2‐ dilinoleyloxy‐ N, N‐dimethyl‐3‐aminopropane (DLin‐DMA) was first developed for siRNA delivery.^[^ ^121^ ^]^ Furthermore, some derivatives, including DLin‐KC2‐DMA and DLin‐MC3‐DMA, were synthesized based on DLin‐DMA to enhance RNA delivery efficacy, among which DLin‐MC3‐DMA also plays a key role in the FDA‐approved siRNA drug Onpattro.^[^ ^122^ ^]^ In addition, MC3 derivate lipids have also been applied to mRNA delivery for protein replacement therapies, antiviral therapies, and cancer therapies.^[^ ^123^, ^124^ ^]^ Polyethyleneimine (PEI) is a commonly used polycation that facilitates the escape of RNAs from endosomes. The amino groups of PEI can undergo protonation at the acidic pH in endosomes, with a protonation degree of ∼20% at physiological pH 7.4 and ∼40% at pH 5.0.^[^ ^125^, ^126^ ^]^ This suggests that PEI is partially protonated under physiological conditions, allowing for an increase in its protonation state within cells. Upon PEI‐RNA complexes entering cells via endocytosis, PEI may act as a “proton sponge” in the acidic endosomal compartments by adsorbing protons. The change in PEI's protonation state within endosomes may also affect the structure of the polyplex, potentially destabilizing it and releasing the RNA.^[^ ^126^ ^]^ However, the non‐degradability of PEI may often lead to cytotoxicity and immune responses. To mitigate these adverse effects, bioresponsive linkages, such as acid‐labile bonds or disulfide bonds, have been incorporated into PEI, enabling biodegradability for less toxic transfection.^[^ ^127^, ^128^, ^129^, ^130^ ^]^

**Figure 4:**
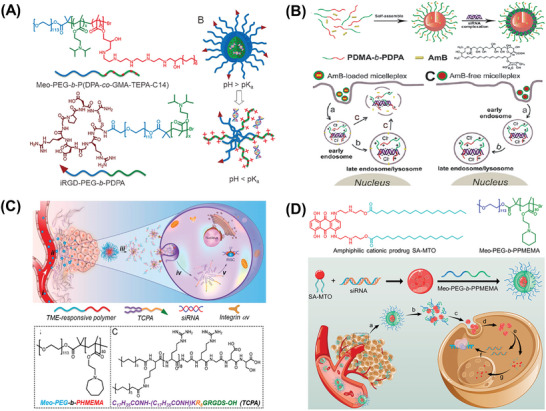
In vivo delivery of RNA by nanocarriers with pH‐responsive protonatable structures in response to weak acid microenvironment. A) Molecular structures of the ultra‐pH‐responsive polymer, Meo‐PEG‐b‐P(DPA‐co‐GMA‐TEPA‐C14) for siRNA delivery. Reproduced with permission.^[^ ^61^ ^]^ Copyright 2016, John Wiley and Sons. B) Schematic diagram of pH‐responsive micelleplexes for siRNA delivery with enhanced siRNA endosomal escape ability. Reproduced with permission.^[^ ^63^ ^]^ Copyright 2011, American Chemical Society. C) pH‐responsive polymer Meo‐PEG‐b‐PHMEMA based NPs for siRNA delivery. Reproduced with permission.^[^ ^131^ ^]^ Copyright 2017, American Chemical Society. D) pH‐Responsive polymer Meo‐PEG‐b‐PPMEMA based NPs for siRNA delivery. Reproduced with permission.^[^ ^64^ ^]^ Copyright 2019, American Chemical Society.

Different from PEI, poly(di‐isopropyl amine ethyl methacrylate) (PDPA) contains tertiary amine structures and shows hydrophobic properties under neutral conditions, which offers good biocompatibility and acid sensitivity that is helpful for endosome escape.^[^ ^62^ ^]^ When NPs formed using PDPA polymer entered the acidic endosome, PDPA underwent the process of protonation, leading to the dissolution of the NPs and the release of relevant RNA drugs^[^ ^61^ ^]^ (**Figure** **4**A). Recently, pH‐responsive diblock copolymers were successfully constructed based on PDPA segments.^[^ ^63^ ^]^ PDMA‐b‐PDPA can self‐assemble to form cationic micelles under neutral physiological conditions. In the micelles, PDPA formed a hydrophobic core to load amphotericin B, whereas the PDMA combined negatively charged siRNA in the PDMA shell. After endocytosis, the siRNA micellar complex consisting of PDPA polymer was protonated and quickly dissociated in the acidic environment of endosomes or lysosomes, releasing the entrapped siRNA into the cytoplasm, thus the siRNA and amphotericin B took effect (Figure 4B). Similar polymers like MeO‐PEG‐b‐PHMEMA^[^ ^131^ ^]^ and MeO‐PEG‐b‐PPMEMA^[^ ^64^ ^]^ were also developed for pH‐responsive delivery of siRNA/ionizable lipid complexes and showed efficient gene silencing ability (Figure 4C,D). In addition, another pH‐responsive core based on poly(β‐amino ester) (PBAE) was developed.^[^ ^67^ ^]^ It was fabricated together with 1,2‐dioleoyl‐3‐trimethylammonium‐propane (DOTAP), 1,2‐distearoyl‐sn‐glycero‐3‐phosphocholine (DSPC), and DSPE‐PEG to form a phospholipid bilayer shell for mRNA delivery. The PBAE core has good responsiveness in an acidic environment, which could allow the lipid‐enveloped PBAE NPs to disassemble to promote endosomal disruption and lead to higher mRNA expression.

The use of protonatable structures that respond to acidic pHs represents a promising approach in RNA/drug delivery. Nevertheless, the choice of suitable protonatable chemical groups that respond specifically to the desired pH range is critical, requiring careful selection and optimization. Moreover, unintended protonation or deprotonation could lead to off‐target effects, underscoring the need for meticulous design and control. Further efforts are still required for the development of this class of nanocarriers for clinical applications.

#### 3.2.2 Acidic pH‐Responsive Degradable Structures

Acid‐degradable chemical linkers, such as imine, ester, acetal/ketal, and maleic acid amide (MAA) derivates,^[^ ^56^ ^]^ are commonly used to develop smart polyplexes with acidic pH‐responsive degradability. These materials consisting of acid‐sensitive chemical bonds are stable under neutral pH conditions, but they can be broken in acidic media; they will undergo structural changes such as charge reversal, size transformation, or morphology transformation under specific pH conditions, resulting in increased cell uptake or deep penetration of tumor tissues.^[^ ^132^ ^]^

**Figure 5:**
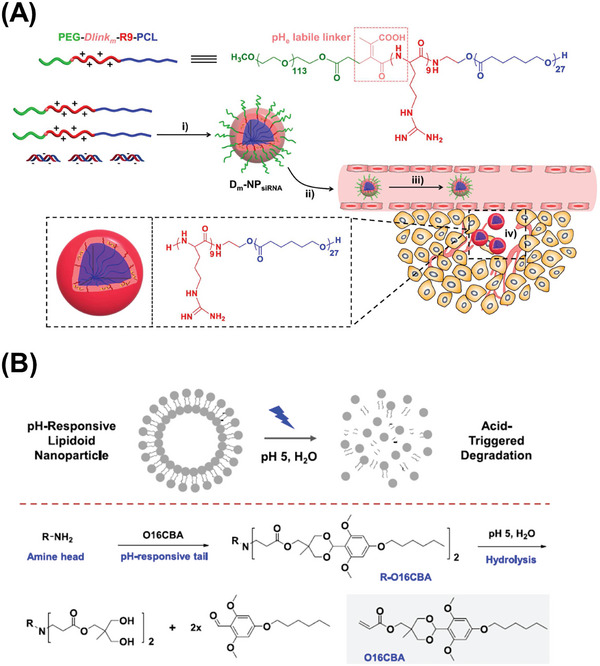
In vivo delivery of RNA by nanocarriers containing pH‐responsive degradable structures in response to weak acid microenvironment. A) PEG‐Dlinkm‐ R9‐PCL‐based siRNA delivery system with a pH labile linker. Reproduced with permission.^[^ ^55^ ^]^ Copyright 2015, American Chemical Society. B) Schematic illustration of the acid‐triggered degradation of lipidoid NPs. Reproduced with permission.^[^ ^59^ ^]^ Copyright 2020, American Chemical Society.

The “acid‐labile copolymer” PEG‐CDM‐*Dlinkm*‐R9‐PCL containing MAA was developed with PEG, 2‐propionic‐3‐methylmaleic anhydride (CDM), and amine‐functionalized poly(ε‐caprolactone)‐R9 (PCL‐R9).^[^ ^55^ ^]^ It can complex siRNA to form stable NPs, and the PEG segments on its surface significantly prolonged the circulation time of siRNA in vivo. Furthermore, after the NPs were accumulated in the tumor by the enhanced permeability and retention (EPR) effect, the acidic microenvironment triggered siRNA NPs’ degradation due to pH responsiveness of 2, 3‐dimethylmaleic acid, which promoted the endosomal escape and significantly improved the silencing efficiency of target genes of siRNA (**Figure** **5**A). More recently, a pH‐responsive nanoplatform comprised of an amphiphilic cationic lipid G0‐C14 and a pH‐liable copolymer (Meo‐PEG‐*Dlinkm*‐PLGA) containing MAA unit was prepared for PTEN mRNA delivery, which could block the activity of PI3K/Akt signaling pathway through PTEN expression and lead to the reversal of trastuzumab resistance in breast cancer treatment.^[^ ^133^ ^]^

In addition, the novel acid‐triggered lipidoid R‐O16CBA containing cyclic benzylidene β‐amino ester group was constructed via the Michael addition reaction with amine head groups and acrylate‐containing O16CBA tails. The lipidiod was then complexed with mRNA and enveloped with DOPE and cholesterol through self‐assembly to form NPs.^[^ ^59^ ^]^ When in an acidic environment, these NPs could undergo a disassociation process triggered by an acetal group of R‐O16CBA, resulting in intracellular mRNA delivery as shown in Figure 5B.

The advancements in acidic pH‐responsive degradable structures have demonstrated potential in the arena of specific RNA delivery, for example to solid tumors. Such delivery materials may also be rapidly cleared in vivo after degradation, thus potentially improving their biocompatibility. Similar to pH‐responsive protonatable groups, the selection of degradable chemical linkers to the desired pH range requires careful consideration, depending on the tissue/cell microenvironment.

### 3.3 ROS‐Responsive Nanocarriers

Reactive oxygen species (ROS) are highly reactive chemicals with high reactivity and strong oxidizing properties, including superoxide anion radical (·O2‐), hydroxyl radical (·OH), and hydrogen peroxide (H_2_O_2_), singlet oxygen (^1^O_2_).^[^ ^134^, ^135^ ^]^ Polyplexes with ROS‐responsive bonds remain stable in the circulatory system, but they can undergo disassociation in oxidative environments such as endosome and cytosome after cellular uptake, which is helpful for RNA to escape from endosome and to be delivered into the cytoplasm.

#### 3.3.1 Thioketal Cross‐Linked Polyplexes

**Figure 6:**
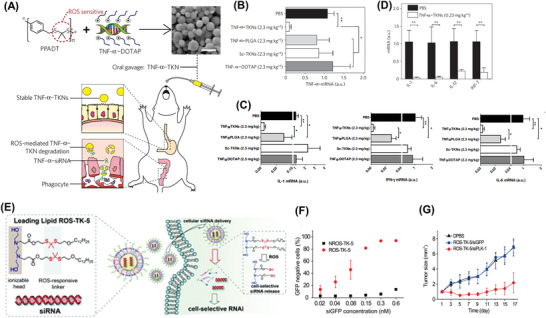
A) Thioketal NPs are formulated from a ROS‐sensitive polymer and release orally delivered siRNA at sites of intestinal inflammation. B) TNF‐α mRNA levels in mice receiving DSS and treated with either scrambled‐ or TNF‐α–siRNA (2.3 mg k^−1^g per day) by means of either TNF‐α–TKNs, TNF‐α–PLGA, Sc–TKNs or TNF‐α–DOTAP. C) Fold change in mRNA of IL‐1, IL‐6, and IFN‐α in the colons of mice receiving DSS and treated with a daily oral gavage of 2.3 mg k^−1^g per day of either TNFα‐siRNA or scrambled siRNA encapsulated within: TNFα‐TKNs, TNFα‐PLGA, Scram TKNs, siRNA‐DOTAP, or PBS, for six days. D) Colonic cytokine mRNA levels in mice receiving DSS and treated with either PBS or TNF‐α–siRNA (0.23 mg siRNA/kg per day) by TKNs. Reproduced with permission.^[^ ^70^ ^]^ Copyright 2010, Nature Publishing Group. E) A ROS‐responsive lipid containing the thioketal group for siRNA delivery. F) The ROS‐TK‐5/siGFP NPs showed a higher GFP knockdown efficiency than that of the NROS‐TK‐5/siGFP NPs. G) Tumor growth curve of HeLa tumor‐bearing mice treated with DPBS, ROS‐TK/siGFP, and ROS‐TK/siPlk‐1 NPs. Reproduced with permission.^[^ ^68^ ^]^ Copyright 2019, Royal Society of Chemistry.

It is common to utilize thioketal bonds for ROS responsiveness.^[^ ^136^ ^]^ Thioketals can readily react with oxidative species and are cleaved into acetone and thiols. Mainly, thioketal bonds exhibit high sensitivity to singlet oxygen and have been widely applied to construct ROS‐sensitive platforms for drug delivery. They can be obtained from the condensation polymerization of 2,2‐dimethoxypropane and dithiol precursors. From this strategy, one polymer composed of thioketal group poly(1,4‐phenyleneacetone dimethylene thioketal) (PPADT) was designed.^[^ ^70^ ^]^ TNF‐α siRNA was first complexed with DOTAP and then hybridized with PPADT to form ROS‐sensitive polyplexes termed thioketal NPs (TNF‐α‐TKNs). It can be delivered orally and disassembled by elevated ROS in the area of intestinal inflammation (**Figure** **6**A). As shown in Figure 6B, mice treated with TNF‐α–TKNs experienced a marked ten‐fold decrease in colonic TNF‐α mRNA. Meanwhile, examination of colonic IL‐6, IL‐1, and IFN‐γ mRNA levels revealed that TNF‐α–TKNs effectively suppressed the activation of these additional proinflammatory signaling pathways by oral gavage (Figure 6C,D). These results imply that orally delivered TKNs perform as well as present systemic delivery systems for siRNA that have been used to treat dextran sodium sulfate (DSS)‐induced colitis. Thioketal linkage has also been utilized in a nanoplatform for siRNA delivery. The group was introduced to the lipid through parallel Michael additions of aliphatic amines and acrylate. This ROS‐responsive lipid ROS‐TK5 could improve the siRNA delivery in ROS‐rich cells via thioketal cleavage^[^ ^68^ ^]^ (Figure 6E). To investigate the ROS responsiveness of the synthetic NPs, ROS‐TK‐5/siGFP was applied to measure the GFP silencing efficiency. GFP‐SiHa cells treated with ROS‐TK‐5/siGFP showed a significant enhancement of the GFP knock down compared to that of the non‐ROS (NROS)‐TK‐5/siGFP complexes (Figure 6F). In vivo anti‐tumor studies also demonstrated significant suppression of the tumor growth treated with ROS‐TK‐5/siPlk‐1 in HeLa tumor‐bearing mice (Figure 6G). In addition to siRNA delivery, thioketals have also recently been used as ROS‐sensitive units to build oligomers *o*‐DHLA for co‐delivery of p53 mRNA and indocyanine green (ICG) to achieve combinatorial mRNA therapy and PDT. Compared to non‐ROS‐responsive mRNA NPs, the *o*‐DHLA mRNA NPs could improve mRNA transfection efficiency. When exposed to the 808 nm irradiation, tumor growth was significantly inhibited by the *o*‐DHLA mRNA/ICG NPs.^[^ ^137^ ^]^

#### 3.3.2 Boronic Esters‐Containing Polymers

**Figure 7:**
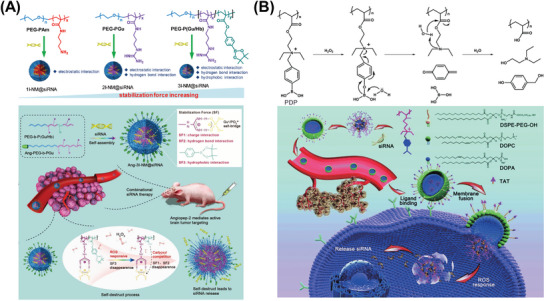
ROS‐responsive materials containing boronic esters for RNA delivery. A) Illustration of formation of Ang‐3I‐NM@siRNA NPs and the mechanisms of ROS‐triggered siRNA release. Reproduced with permission.^[^ ^72^ ^]^ Copyright 2019, John Wiley and Sons. B) ROS‐responsive mechanism of PDA structure and a schematic illustration of lipid‐coated ROS‐responsive NPs for siRNA delivery. Reproduced with permission.^[^ ^73^ ^]^ Copyright 2017, Royal Society of Chemistry.

Aryl boronic acid and ester bonds can be selectively degraded by H_2_O_2_, producing phenol and boronic acid as oxidation products. They are susceptible to H_2_O_2_, but may not be oxidized by other ROS,^[^ ^136^ ^]^ and are thus important building blocks for constructing H_2_O_2_‐selective polymers. Boronic esters were introduced into *N*‐(3‐methacrylamidopropyl) guanidinium (Gu) and MeO‐PEG‐CPADN to obtain the polymer PEG‐b‐P(Gu/Hb) by RAFT polymerization. Another chain Ang‐PEG‐*b*‐PGu targeting polymer containing boronic esters was also prepared by RAFT reaction.^[^ ^72^ ^]^ When siRNA was encapsulated in PEG‐b‐P(Gu/Hb) and Ang‐PEG‐*b*‐PGu to form Ang‐3I‐NM@siRNA NPs, it was easily released in glioblastoma because of ROS‐triggered degradation of the nanoplatform (**Figure** **7**A). A similar strategy was also applied using phenylboronic acid to synthesize the ROS‐responsive polymer PDP.^[^ ^73^ ^]^ siRNA was complexed with PDP through electrostatic interaction. The siRNA/PDP complex was further coated with a layer of a lipid envelope consisting of 1,2‐Dioleoyl‐sn‐glycero‐3‐phosphate (DOPA), 1,2‐dioleoyl‐sn‐glycero‐3‐phosphocholine (DOPC), non‐fouling PEG and a cell‐penetrating peptide (TAT), which effectively fused with the cell membrane, with the goal of improving the efficiency of cellular uptake as well as avoiding endo/lysosomal trapping (Figure 7B).

#### 3.3.3 Diselenide‐Containing Polymers

Diselenide can exert excellent activity in response to either oxidants or reductants, which makes it a promising candidate for dual redox response. In a study, the ROS‐degradable cationic polymer was developed based on PEI. First, the responsive polycation Se‐PEI was synthesized via cross‐linking of PEI and ROS linkage diselenide bonds.^[^ ^75^ ^]^ After that, carboxylated mannan (Man‐COOH) was coated with Se‐PEI in order to increase the targeting ability of macrophages. The resulting polyplex‐encapsulated TNF‐α siRNA could easily reach macrophages, thus triggering the degradation of Se‐PEI to enhance intracellular siRNA release, which was an ideal polyplex for systemic delivery of siRNA to treat hepatic inflammation in mice with acute liver failure (ALF). Another study developed a platform called UCNOs (UCNPs‐PEIRB‐PEISeSe/siRNA‐R8‐HA), which were composed of upconversion nanoparticles (UCNPs) core functionalized with an inner coating layer of photosensitizer rose bengal (RB) conjugated PEI 600, a middle coating layer of singlet oxygen‐sensitive diselenide linked PEI 600 with therapeutic siRNA and cell‐penetrating peptide R8 modification, and an outer coating layer of negatively charged hyaluronic acid (HA).^[^ ^76^ ^]^ Upon the irradiation at 808 nm, the UCNPs core generates emissions ∼540 nm, which activated RB to boost ROS generation for PEI‐SeSe decomposition, inducing a fast and efficient siRNA release for improved gene silencing in vitro and anti‐tumor effect in vivo.

Given the distinct levels of ROS between normal and diseased tissues, ROS‐responsive systems could considerably improve drug/RNA delivery efficiency. Despite the enormous preclinical studies in this specific area, several factors should be considered. First, the type of ROS is important for the selection of delivery vehicles. For instance, thioketal linders could be cleaved in the presence of ^1^O2, which is a major type of ROS generated by different cell types, especially neutrophil granulocytes.^[^ ^138^ ^]^ This suggests that thioketal‐responsive RNA NPs may be used for disorders caused by neutrophil granulocytes. For H_2_O_2_, it is mainly generated by superoxide dismutase enzymes (SOD1 and SOD2) in mitochondria.^[^ ^139^ ^]^ Boronic ester linkers are sensitive to H_2_O_2_, which offers the opportunity to build PBA‐based NPs for RNA delivery to mitochondria‐related diseases. Another consideration is the premature, delayed, or incomplete ROS‐responsive release of RNA, which may jeopardize its therapeutic effect. Beyond endogenous ROS, it is also possible to combine ROS‐responsive delivery with other strategies (such as PDT) that can promote ROS regeneration in target locations to further boost RNA release, thus achieving enhanced therapeutic efficiency.

### 3.4 Temperature‐Responsive Nanocarriers

Normal human organs have a temperature ranging from 36 to 38 °C. For instance, the stomach has a temperature of 36.5–37.5 °C; the spleen and kidney have a temperature of ∼37 °C. A tumor is an abnormal mass of tissue that forms when cells grow and divide out of control, meaning that the tumor can produce pyrogens, a substance that can cause an infection. When an infection occurs, the immune system generates more heat to eliminate the bacteria or germs in the area, which means that the abnormal tissue like tumor or inflammation area will have a slightly higher temperature, ranging from 38 to 42 °C. With these temperature differences, nanovehicles that respond to the temperature can be used as tumor site‐specific nanotransporters. The most widely used temperature‐sensitive bonds are probably N‐isopropylacrylamide (NIPAAM), which could be responsive to temperature change through hydrophilic and hydrophobic alternation.

Poly‐NIPAAM (PNIPAAM) exhibits a distinct nonlinear solubility‐temperature relationship, as manifested by a pronounced thermotropic transition between soluble and insoluble states.^[^ ^140^ ^]^ This phase transition temperature, also referred to as the cloud point or lower critical solution temperature (LCST), delineates a transition from a polymer's hydrated state (attributed to the formation of water‐polymer hydrogen bonds) to a dehydrated state. In the latter, the entropic preference disrupts these hydrogen bonds, instigating polymer collapse or macroscopic phase separation, contingent on polymer concentration.^[^ ^87^, ^141^, ^142^ ^]^ PNIPAAM can undergo hydrophobic aggregation at temperatures surpassing 32 °C, with the specific temperature varying based on concentration and molecular weight.^[^ ^143^ ^]^ The LCST of the polymer could be strategically modulated through copolymerization with selected hydrophilic or hydrophobic blocks for tailored applications.^[^ ^144^ ^]^ Specifically, copolymerization with hydrophilic units, yielding a phase transition near 37 °C, has proven beneficial due to the polymer's sensitivity to physiological temperatures. Consequently, complexes of these copolymers with nucleic acids, which can be formed at sub‐cloud points, are anticipated to exhibit nucleic acid release within human cells or bodily fluids, driven by the polymer's structural collapse at physiological conditions.^[^ ^145^ ^]^ Such intricacies in polymer design, achieved by introducing different hydrophilic or hydrophobic blocks into PNIPAAM, may enable targeted RNA delivery at precise temperatures.

**Figure 8:**
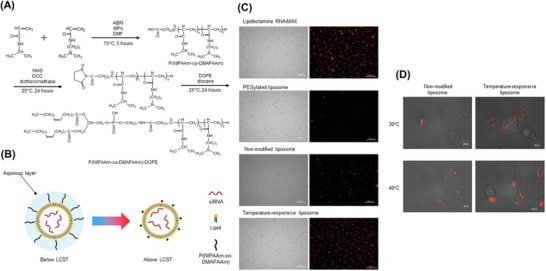
A) Chemical structure of temperature‐responsive polymer P(NIPAAm‐co‐DMAPAAm)‐DOPE. B) Illustration of a temperature‐responsive polymer‐modified liposome as a siRNA carrier. C,D) Cellular association of Alexa fluor 555‐labeled siRNA liposomes with each formulation after incubation with HeLa cells for 2 h at 37 °C or 30 °C versus 40 °C, observed by fluorescence microscopy. Left panel is a bright field and the right panel is fluorescence in (C). Reproduced with permission.^[^ ^89^ ^]^ Copyright 2017, Elsevier Ltd.

Temperature‐responsive copolymers containing units of NIPAAM and (3‐acrylamidopropyl) trimethylammonium chloride (AMPTMA) were synthesized for siRNA delivery.^[^ ^87^ ^]^ AMPTMA units of this polymer are prone to efficiently complex negatively charged siRNA, while the NIPAAM part is responsive to temperature, thus producing the ability of these copolymers to release siRNA into human fibrosarcoma cells. This system could deliver siRNAs efficiently, specifically and without presenting relevant cytotoxicity. Another temperature‐responsive polymer P(NIPAAm‐co‐DMAPAAm) containing a NIPAAM unit was also constructed^[^ ^89^ ^]^ (**Figure** **8**A). This temperature‐responsive polymer‐modified liposome could induce rapid intracellular siRNA delivery because P(NIPAAm‐co‐DMAPAAm) exhibited a lower critical solution temperature (LCST), changing its nature from hydrophilic to hydrophobic above the LCST, which enhanced cellular uptake of the lipoplexes for gene silencing (Figure 8B). The cellular association of each siRNA liposome formulation was further examined using fluorescence microscopy. The level of fluorescence from the cells at the temperature of 37 °C was found to follow the order: temperature‐responsive liposomes = RNAiMAX > non‐modified liposomes > PEGylated liposomes, as shown in Figure 8C. The cellular uptake at different temperatures (30 °C vs 40 °C) was also investigated (Figure 8D). At 30 °C, the temperature‐responsive liposomes exhibited lower cellular association similar to that of non‐modified liposomes, with red fluorescent dots of AF‐siRNA surrounding the cell membrane. In comparison, a higher number of red fluorescent dots representing the temperature‐responsive liposomes were observed in the cytoplasm compared to non‐modified liposomes at 40 °C, suggesting more cellular uptake of the temperature‐responsive system.

Temperature‐responsive nanocarriers present a potentially viable avenue for targeted delivery of RNAs, capitalizing on the unique temperature characteristics of different tissues, particularly inflamed or tumorous tissues. However, temperature‐responsive materials have limitations that need to be addressed. One such limitation is the inadequate control over the thermal triggering of certain nanocarriers. Another one is that the biodegradability of pristine PNIPAAM is not deemed satisfactory, thereby restricting its potential biomedical applications. The enhancement of biodegradability of these materials may be essential in future development efforts.

### 3.5 Enzyme‐Responsive Nanocarriers

The occurrence and development of a tumor and the overexpression of related genes in the tumor will lead to changes in the expression and activity of some enzymes in the internal and external environment of tumor cells, which provides a new method for the design of enzyme‐responsive RNA delivery vectors. Compared with other stimulus conditions, most enzymatic reactions are fast, efficient, and highly specific. At present, ester bonds cleavable by esterase or short peptides cleavable by protease are commonly used to modify nanomedicine carriers, to achieve controlled release and accumulation of RNA at specific biological targets. Among them, matrix metalloproteinase (MMP) or cathepsin has been widely explored in the design of enzyme‐responsive RNA nanocarriers.

**Figure 9:**
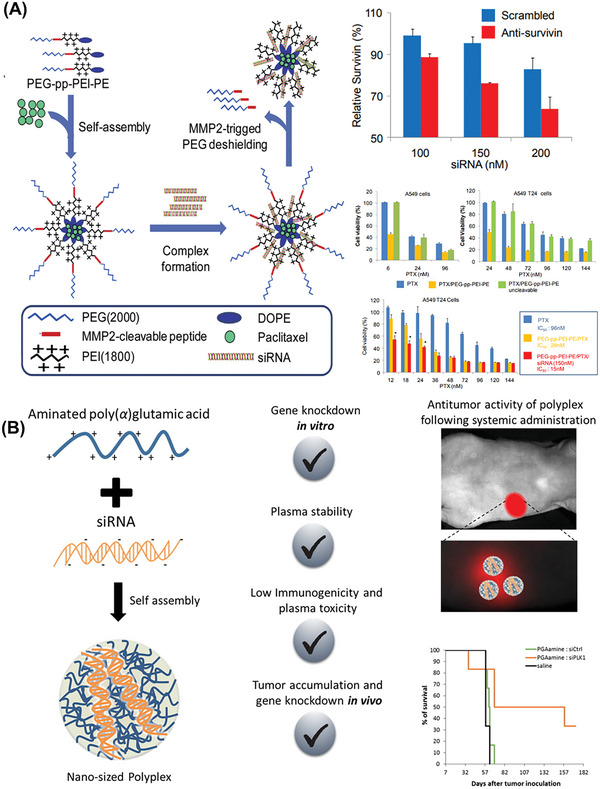
A) RNA delivery strategies based on matrix metalloproteinase‐2 (MMP‐2) triggered PEG de‐shielding. Reproduced with permission from Ref. [90]. Copyright 2014 Elsevier. B) Cathepsin B biodegradable nanocarriers for siRNA delivery. Reproduced with permission.^[^ ^93^ ^]^ Copyright 2017, Elsevier.

Matrix metalloproteinase is a protease involved in the degradation of tumor extracellular matrix. Compared with normal tissues, it is relatively highly expressed in almost all types of tumors. A multifunctional polymeric micelle containing MMP‐2 sensitive peptide for tumor‐targeted co‐delivery of siRNA (si‐survivin) and hydrophobic chemotherapy drugs was designed to enhance the uptake of siRNA and paclitaxel in tumor cells. MMP‐2 sensitive peptide (GPLGIAGQ) was used as the linking arm between the cationic polymer PEI and PEG to synthesize an amphiphilic MMP‐2 sensitive material PEG‐pp‐PEI‐PE.^[^ ^90^ ^]^ First, PEG‐pp‐PEI‐PE encapsulated the hydrophobic chemotherapeutic drug paclitaxel (PTX) and then loaded negatively charged siRNA through electrostatic interaction to form micelles. When it reached tumor tissue with over‐expression of MMP‐2, the sensitive peptide GPLGIAGQ was cleaved by MMP‐2 response, then PEG was removed to leave the micelles with positive charges, thereby increasing the uptake of siRNA by tumor cells, enhancing the ability to escape from endosomes and promoting anti‐tumor efficacy (**Figure** **9**A). In vitro studies showed the survivin protein was down‐regulated for ∼30% at 150 nm survivin siRNA and the silencing effect was dose‐dependent. Incubation of PEG‐pp‐PEI‐PE/PTX with A549 or A549‐T24 cells significantly increased the cytotoxicity of PTX compared to those of free PTX or its nonsensitive micelles, while the co‐administration of survivin siRNA and PTX resulted in a substantial reduction in the IC50 of PTX, lowering it to ∼15 nm. Altogether, this enhanced antitumor activity was a result of the enhanced co‐delivery efficiency and synergistic effect of PTX and survivin siRNA.

Many tumor tissues exhibit high expression of cathepsin, and various cathepsin‐based vectors have been prepared for tumor‐targeted delivery of RNA drugs.^[^ ^146^ ^]^ Polyglutamic acid (PGA) is a synthetic water‐soluble polymer that can be degraded by cathepsin without immunogenicity or toxicity. A siRNA delivery material with PGA fragments was synthesized, which was responsively degraded by cathepsin and showed positive therapeutic effects in treating ovarian cancer.^[^ ^93^ ^]^ In this study, the carboxyl group in PGA was modified to a positively charged amino group, which made it capable of loading negatively charged siRNA. This PGA derivative complexed siRNA through electrostatic interaction and formed a stable carrier system in plasma to protect siRNA from enzymatic degradation. When NPs accumulated in tumor tissues through the EPR effect and were ingested by tumor cells, PGA degraded under the action of cathepsin, thus releasing siRNA and exerting its anti‐tumor efficacy (Figure 9B).

### 3.6 Hypoxia‐Responsive Nanocarriers

Hypoxia is a common phenomenon in malignant tumors. Its occurrence is mainly related to the unrestricted growth of tumors, increased oxygen consumption, insufficient blood supply, and vascular dysplasia of tumor tissues. It is also a pathological process associated with many clinical diseases. The tumor area is the most common hypoxic environment because it possesses the following properties: uncontrollable cell proliferation, altered metabolism, abnormal tumor blood vessels, and overexpression of some reductases (such as nitro‐reductase and azo reductase), resulting in a decrease in the oxygen delivery to the tumor area. In addition, hypoxia can result in acidification and increased oxidative stress, with profound consequences for cell physiology and tumorigenesis,^[^ ^147^ ^]^ and hypoxia could also increase ROS production, thus leading to cell death.^[^ ^148^, ^149^ ^]^ The differences in oxygen levels between normal human organs and abnormal tissues like tumors allow site‐specific NPs to deliver RNAs to these abnormal areas. The hypoxia‐responsive groups are used as the recognition unit of hypoxia‐responsive materials, which can react with biological reductase in cells. The materials for RNA delivery usually contain a NO_2_ group or N=N group; when they are placed in a hypoxic environment, these two groups undergo chemical changes to NH_2_, and the reduced NPs become a loose structure. Due to the displacement of the anions, they fall apart and then release the RNA.^[^ ^150^ ^]^ Therefore, polymer materials consisting of nitro or azo groups are widely used in the construction of hypoxia‐response nanocarriers to increase RNA uptake by target cells.

**Figure 10:**
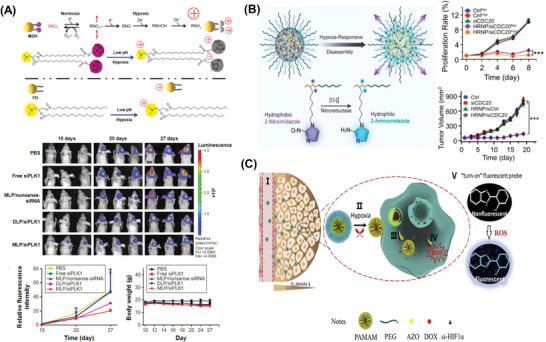
Hypoxia‐responsive RNA drug delivery systems based on nitroimidazole (A) and (B). A) Reproduced with permission.^[^ ^83^ ^]^ Copyright 2020, American Chemical Society. B) Reproduced with permission.^[^ ^83^, ^84^ ^]^ Copyright 2017, Dove Medical Press Ltd. Hypoxia‐responsive RNA drug delivery systems based on (C) azobenzene. Reproduced with permission.^[^ ^86^ ^]^ Copyright 2018, Elsevier.

A new lipid molecule was designed containing tertiary amine as the hydrophilic head and hexadecyl chains with nitroimidazole groups as the hydrophobic tail.^[^ ^84^ ^]^ The nanocarrier consisting of the aforementioned lipid, cholesterol, and DSPE‐PEG was used to encapsulate siPLK1. When the nanocarrier reaches the tumor site, the weak acid microenvironment of the tumor tissue protonates the tertiary amino group, giving it a positive charge. At the same time, the hypoxic environment of the tumor tissue also converted the hydrophobic nitroimidazole group into positively charged aminoimidazole, which greatly increases the uptake of siPLK1 by tumor cells, significantly enhancing gene silencing and inhibiting tumor growth (**Figure** **10**A). In addition, the hypoxia‐responsive nanoplatform was constructed using a nitroimidazole group and cationic lipid for siCDC20 delivery.^[^ ^83^ ^]^ Upon reaching the hypoxic tumor environment, the nitroimidazole group changed to aminoimidazole, leading to the dissociation of the nanocarrier system and rapid release of siRNA. The effect of CDC20 silencing on MCF‐7cell proliferation was measured using this hypoxia‐responsive nanoplatform (HRNP‐siCDC20). Remarkable inhibition of cell growth by HRNP/siCDC20 was detected in MCF‐7 cells compared to the untreated group, and the cells showed the highest inhibition rate under hypoxic conditions (Figure 10B). Moreover, HRNP/siCDC20 demonstrated the most significant inhibition of tumor growth on breast tumor models, as compared to other groups.

Azobenzene, another hypoxia‐responsive group, was introduced to the novel lipid PEG‐AZo‐PEI‐DOPE.^[^ ^85^ ^]^ It was constructed via azobenzene, PEG, PEI, and DOPE. When it was complexed with siRNA, the PEG segments on the surface of the carrier significantly prolonged the circulation time of siRNA in vivo. A similar strategy based on azobenzene was also used to develop hypoxia‐responsive NPs for siHIF‐1α.^[^ ^86^ ^]^ When the system enters the tumor tissue and the hypoxic microenvironment, the breakage of the azobenzene (AZO) structure caused the PEG group to detach from the surface of the PAMAM, which was conducive to the penetration of NPs into tumor tissue and the uptake by tumor cells. Based on the azobenzene group, the polyamidoamine (PAMAM) dendrimer was conjugated to PEG_2000_ using a hypoxia‐sensitive linker, azobenzene, to form PAMAM‐AZO‐PEG (PAP) (Figure 10C). siHIF‐1α was adsorbed to PAMAM through electrostatic interaction, and DOX was loaded in the hydrophobic core of PAMAM to form a compact NP. siRNA was released rapidly when the NPs entered the hypoxic tumor environment.

### 3.7 ATP‐Responsive Nanocarriers

Adenosine triphosphate (ATP) is a crucial biomolecule involved in cellular energy metabolism. ATP is present in low concentrations (<0.4 mm) in the extracellular environment but is relatively concentrated intracellularly (1–10 mm) in healthy tissues^[^ ^151^, ^152^ ^]^; while in the diseased tissues like tumor environment, the concentrations of extracellular ATP can reach levels as high as several hundred micromolar.^[^ ^153^, ^154^ ^]^ The prominent difference in the ATP level between the healthy and diseased tissues provides a biological rationale for the design of ATP‐mediated drug release systems,^[^ ^155^ ^]^ as well as motivates the development of RNA delivery vehicles that respond to ATP levels.

**Figure 11:**
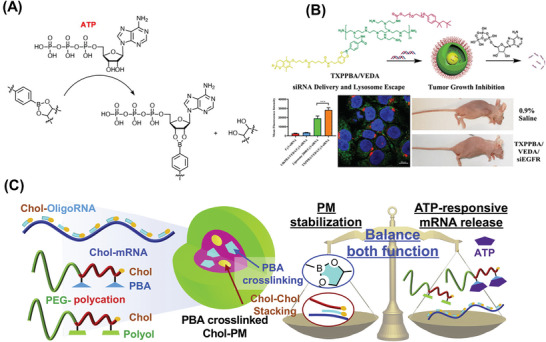
ATP responsive systems for RNA delivery. A) The ATP‐responsive mechanism of phenylborate; B) Developed polymer TXPPBA/VEDA and the formed NPs for siRNA delivery. Reproduced with permission.^[^ ^94^ ^]^ Copyright 2019, American Chemical Society. C) ATP‐responsive polyplex micelles with optimal density of phenylboronate ester for mRNA delivery. Reproduced with permission.^[^ ^96^ ^]^ Copyright 2021, Elsevier Ltd.

The ribose in the ATP structure has a strong affinity for phenylboronic acid (PBA) (**Figure** **11**A), which can break the phenylborate structure through competitive binding.^[^ ^156^, ^157^ ^]^ Therefore, various RNA drug delivery vehicles with phenylborate structures have been designed for ATP response in tumor cells.^[^ ^95^, ^158^ ^]^ The ATP‐responsive siRNA delivery vehicle containing a phenylborate structure was developed to accelerate the release of siRNA in the cytoplasm in response to the cleavage of ATP.^[^ ^94^ ^]^ The main structure of the carrier was composed of the modified PEI polymer conjugated with 4‐carboxyphenylboronic acid and dopamine‐grafted vitamin E (VEDA) to obtain cationic polymer TXPPBA/VEDA. When the modified TXPPBA/VEDA/siRNA complex entered the tumor cells, the boronate bond became cleavable due to a high level of ATP, thereby making the NPs disassemble and increasing the release of the entrapped siRNA to improve silencing efficiency (Figure 11B). Similarly designed ATP‐responsive mRNA‐loaded polyplex micelles crosslinked with the phenylborate esters were reported for mRNA delivery in tumor cells (Figure 11C).^[^ ^96^ ^]^ PEGylated block polymers derived from phenylboric acid and polyol groups were synthesized for responsive delivery of mRNA.^[^ ^96^ ^]^ First, the negatively charged mRNA combined with the polycation fragment via electrostatic interaction to form a polymer/mRNA complex. Then the structure of phenylboric acid and polyol in the material formed a phenylborate ester via a crosslink reaction to improve the stability of the polymer/mRNA complex. When tumor cells endocytosed the complex, the ribose structure in ATP could competitively bind to the phenylborate group, resulting in the cleavage of the phenylborate ester bond and the responsive release of mRNA. Compared to redox, pH, enzyme, and hypoxia stimuli, the use of ATP for responsive RNA delivery has been relatively limited. This may be due to the lack of ATP‐responsive materials.

## 4 External Stimuli‐Responsive Systems for RNA Delivery

To date, great effort has been put into the development of external stimuli‐responsive drug delivery systems.^[^ ^111^, ^159^ ^]^ They offer the potential advantage of overcoming patient‐to‐patient biomedical external factors that only directly control the drug release.^[^ ^160^ ^]^ Furthermore, when compared to internal stimuli triggered therapeutics, external stimuli are endowed with more feasibility in a disciplined manner during treatment and can be manually controlled and manipulated based on individual requirements.^[^ ^161^ ^]^ Several external stimulants have been preliminarily explored in RNA nanotherapeutics delivery, such as light, ultrasound (US), and magnetic field. Herein, we discuss representative external stimuli‐responsive nanocarriers for RNA delivery.

### 4.1 Light‐Responsive Nanocarriers

Light possesses the advantages of noninvasiveness and high controllability, which makes it the most convenient tool commonly used as the external stimulus to control and modulate the drug release process in vitro and in vivo. Under illumination, the structure of photosensitive nanocarriers directly or indirectly undergoes transformation to release loaded drugs. However, UV light is usually applied for topical treatments due to the poor penetration (<1 cm) of radiation below 650 nm. On the other hand, near‐infrared (NIR) laser (650–900 nm) can have deeper penetration (∼10 cm) and result in minimal tissue damage.^[^ ^160^, ^161^ ^]^ Till now, several strategies have been used to produce light‐activated delivery systems: degradation of nanocarrier (e.g., o‐nitrobenzyl moiety) caused by light reaction, morphological transformation (e.g., azobenzene moiety) of nanocarrier induced by photoisomerization, and photothermally (temperature) or photodynamically (ROS) induced nanocarrier rupture.

**Figure 12:**
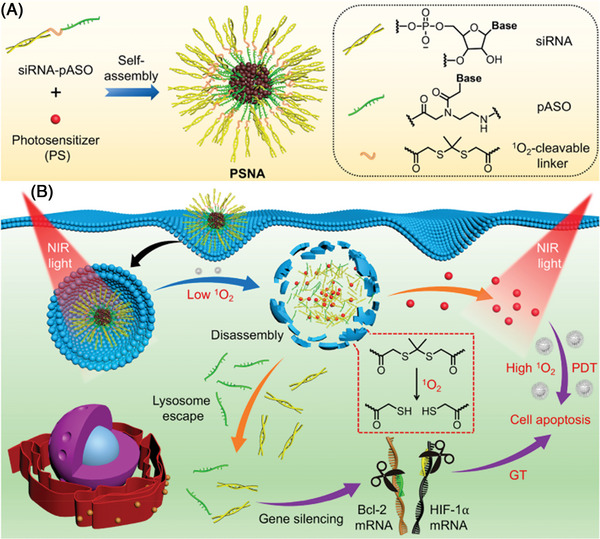
A) Illustration of the formulation of PSNA. B) Schematic representation of the use of PSNA to deliver siRNA, pASO, and PS for combination cancer therapy. Reproduced with permission.^[^ ^164^ ^]^ Copyright 2021, American Chemical Society.

Light‐responsive linkage is commonly used because it can be remotely applied with high spatial and temporal precision and has been widely used as an external stimulus to control cargo release from delivery systems. Nitrobenzyl (NB) bonds are photoreaction‐induced degradation groups commonly used to construct light‐responsive nanosystems. For instance, an amphiphile contains a long hydrophobic tail and a cationic head conjugated with a photolabile NB ester bond, which was built to encapsulate siRNA.^[^ ^162^ ^]^ When exposed to UV light irradiation, cleavage of the NB bond caused detachment of the hydrophilic shell and promoted siRNA release from the complex to improve gene silencing efficiency eventually. In early studies, Si‐UCNPs were developed and then conjugated using a photoactive o‐nitrobenzyl ester functionalized thiol‐linker with a positively charged alkyl amine at the end of the structure. Upon NIR irradiation, the UCNPs emitted UV light, and then o‐nitrobenzyl linker was cleaved, thus promoting siRNA release to silence target gene expression in living cells.^[^ ^163^ ^]^ Recent studies demonstrated that if the photosensitizer (PS)‐containing NPs are irradiated with NIR light for a short time, the PS could generate ROS and induce NP degradation to release drugs. Therefore, a photo‐labile spherical nucleic acid (PSNA) for carrier‐free and NIR photo‐controlled self‐delivery of siRNA and ASO was constructed. PSNA was self‐assembled from an amphiphilic oligonucleotide drug (OND) conjugate composed of a hydrophilic siRNA linked with a hydrophobic peptide nucleic acid‐based ASO (pASO) via a ROS‐cleavable linker (**Figure** **12**). Upon NIR irradiation, the PS could generate ROS, which induced the disassembly of the nanoplatform, thus promoting the lysosomal escape of the released siRNA and pASO.^[^ ^164^ ^]^

While a number of photoresponsive systems have been developed with the potential to enable spatiotemporal control of RNA delivery, one primary drawback of the light‐triggered strategy remains the limited tissue penetration depth with light. Further efforts should also address the biosafety concern, as the utilization of light‐based photothermal/photodynamic methods to trigger RNA release may potentially produce an excessive amount of heat or singlet oxygen, leading to DNA damage or other side effects.

### 4.2 Ultrasound‐Responsive Nanocarriers

Ultrasound (US) is a non‐invasive stimulus with several beneficial features for triggered drug/gene delivery, including deep penetration into the body, precise application, excellent spatiotemporal control, and ease of control. It has been extensively applied for the diagnosis and treatment of various diseases. US‐responsive drug delivery systems have obtained much attention and become an attractive goal in anticancer therapy,^[^ ^165^ ^]^ as they can facilitate the transport of cell membrane‐impermeable compounds into living cells by temporally inducing cell membrane openings.

Microbubbles consist of gas‐filled cores and stabilized shells, which can amplify the biophysical effects of US by cavitation. In addition to their clinical application as contrast agents in US imaging, microbubbles have also been extensively studied as carriers for drug and gene delivery. In a previous study, US‐responsive microbubbles loaded with mRNA‐lipoplexes were developed.^[^ ^166^ ^]^ The aim was to explore a physical approach for delivering mRNA to the cytosol of dendritic cells (DCs). Successful uptake of mRNA‐lipoplexes by DCs was observed, leading to a notable expression of luciferase and EGFP by the DCs following US‐guided transfection. Importantly, this transfection method did not compromise the viability or maturation capacities of the DCs. In another study, C3F8 gas as the core and phospholipids as the outer layer were employed to fabricate nanobubbles.^[^ ^167^ ^]^ These nanobubbles were utilized for the targeted delivery of isocitrate dehydrogenase 1 (IDH1)‐siRNA to gliomas. The results demonstrated that the nanobubbles loaded with siRNA effectively disrupted the expression of IDH1 both in vitro and in vivo when subjected to ultrasound sonication, leading to the increase of siRNA uptake and inhibition of tumor growth. siRNA‐encapsulated US‐responsive microbubbles were also developed from polymeric siRNA micelles and liposomal microbubbles using a hetero‐assembling strategy. By subjecting the tumor site to low‐frequency US at 1 MHz following intratumoral injection of XIAP siRNA microbubbles, the tumor tissue permeability was enhanced, allowing for improved delivery of siRNA into the deeper regions of the tumor. This resulted in a significant enhancement in the silencing of the XIAP gene and activation of cleaved caspase‐3, and ultimately a favorable therapeutic effect on a human cervical cancer xenograft model in nude mice.^[^ ^168^ ^]^ US‐responsive nanobubbles were also used to encapsulate both the anticancer drug paclitaxel and siRNA by hetero‐assembly of polymeric micelles and liposomes. When these nanobubbles were exposed to ultrasound, effective tumor‐penetrating codelivery of siRNA and paclitaxel was achieved.^[^ ^169^ ^]^

### 4.3 Magnetic‐Responsive Nanocarriers

Magnetic fields can act on nucleic acid delivery vectors associated with magnetic NPs, direct vectors toward target cells, and achieve effective nucleic acid delivery. Magnetic iron oxide can be straightforwardly complexed with RNA after surface modification. In particular, magnetic NPs modified with PEI can effectively adsorb double‐stranded RNA through electrostatic interaction, not only inhibiting the migration of glioma cells but also showing the superiority of high‐contrast MRI imaging. Recently, cationic magnetic nanocomposites (MNCs) composed of branched PEI‐coated iron oxide NPs (IONPs) were successfully synthesized, which could penetrate the cell wall and deliver miRNA‐34a into cancer cells.^[^ ^170^ ^]^ Besides, the magnetically responsive hybrid NPs comprising superparamagnetic iron oxide NPs (SPIONs) coated with calcium phosphate (CaP) and PEG‐polyanion block copolymers were prepared for siRNA delivery to breast cancer cells. In this nanoplatform, SPIONs acted as a magnetically responsive carrier while CaP served as a middle layer to protect SPIONs and adsorb siRNA. These magnetic‐responsive NPs exhibited great potential for cancer therapy based on the RNAi effect, plus a magnetic capability to modulate and enhance siRNA accumulation in the target tissue.^[^ ^171^ ^]^

In addition, a dual pH‐ and magnetic field‐responsive nanocarrier has been developed for experimental use by synthesizing a pH‐sensitive and vitamin A (VA)‐conjugated copolymer, VA–polyethylene glycol–polyethyleneimine–poly(N‐(N’,N’‐diisopropylaminoethyl)‐co‐benzylamino) aspartamide (T‐PBP). This copolymer was assembled into superparamagnetic iron oxide‐decorated cationic micelles for miRNA delivery. These T‐PBP micelles effectively transported miRNA‐29b and miRNA‐122 to hepatic stellate cells in liver fibrotic rats. The miRNA micelles resulted in a synergistic anti‐fibrosis effect by downregulating the expression of genes related to fibrosis.^[^ ^172^ ^]^ In another case, a siRNA‐CPPs conjugate (siRNA‐CPPs) was loaded into thermal and magnetic dual‐responsive liposomes (siRNA‐CPPs/TML).^[^ ^173^ ^]^ The siRNA‐CPPs/TML formulation demonstrated superior efficacy in terms of targeted delivery, antitumor activity, and gene silencing efficiency in a murine model of MCF‐7 xenograft.

Although the proof‐of‐concept of magnetic responsive RNA delivery has been demonstrated, a significant challenge lies in the complexity of such delivery systems, which typically consist of IONPs and cationic materials. This may raise concerns regarding future scale‐up and potential toxicity. The development of more robust and biocompatible platforms would be needed in future research.

## 5 Conclusion and Future Prospects

RNA‐based therapeutics have emerged as a potent strategy for treating various diseases, and their combination with other therapies (e.g., phototherapy, immunotherapy, and chemotherapy) has also been employed in the biomedical field.^[^ ^36^, ^38^, ^39^, ^174^, ^175^, ^176^, ^177^, ^178^, ^179^ ^]^ Although the design and chemical modification of RNAs has achieved adequate stability, efficacy, and specificity, successful RNA therapeutics may still be highly dependent upon the development of versatile and powerful delivery carriers. This review has considered the barriers in the RNA therapy field and the current solutions offered by nanocarrier delivery systems. Specific stimuli‐responsive nanocarriers have been developed for more accurate and efficient RNA delivery to overcome the difficulties in traditional RNA delivery nanotechnologies. The choice of stimuli‐responsive RNA delivery may depend on various factors, such as the intended application and specific tissue environment. The overall desire to develop stimuli‐responsive RNA delivery NPs, as compared to non‐responsive NPs, is to further improve RNA bioactivity and tissue/cell selectivity (thus reducing potential off‐target effects and systemic toxicities). Given that most RNA molecules are highly hydrophilic and negatively charged, they can generally complex with ionizable/cationic materials through charge interactions to form NPs, or in some cases, even with neutral/negative materials through other types of interactions. Thus, from this perspective, stimuli‐responsive NPs may be generally applicable to RNA molecules with different molecular weights (e.g., siRNA and mRNA). The selection of stimuli would then largely depend on the indication and diseased tissue. Different stimuli could also be used for responsive RNA delivery to one type of tissue (e.g., solid tumor), as discussed above. Similarly, various stimuli‐responsive NPs, such as lipid‐ and polymer‐based NPs, have been reported for delivery of the same type of RNAs. It should be noted that the formulation of NPs will need to be tuned/re‐optimized (or even re‐designed) for different RNA types, particularly those with large size differences.

The engineering of bio‐responsive moieties into nanocarrier structures enables the site‐specific alteration of the carrier properties in response to various biological stimuli. By taking advantage of the unique physiological microenvironment of tissues/cells, bio‐responsive nanocarriers could effectively overcome the problems of poor stability, low tissue accumulation, limited endosomal escape ability, and insufficient protein alteration efficiency associated with RNA delivery in vivo. Although the internal stimuli‐responsive RNA delivery strategy has advanced significantly in terms of design and application, some critical issues remain. First, we need to gain a deeper understanding of how the RNA carrier system interacts with biological components in vivo. Second, owing to the complexity of the human physiological system, some stimulus‐response circumstances may exhibit non‐specific distributions. For instance, specific normal cells or non‐tumor tissues may also have a low pH, elevated GSH concentrations, or overexpression of particular enzymes, all of which may result in non‐specific responses or inadequate stimulus sensitivity of the carrier system. Notably, biological characteristics may vary among patients, which could lead to the heterogeneous efficacy of the stimulus responses. In comparison, the major disadvantage of external stimuli‐responsive NPs for RNA delivery is that they require remote devices to trigger, which is not convenient. In addition, some treatments involving light treatment or strong magnetic fields may only be practical in clinics. Another shortcoming of some external stimuli‐responsive NPs is the depth of penetration needed for the payloads' release‐ from the NPs.

The complexities of stimuli‐responsive RNA carrier structures may also complicate large‐scale manufacture, thus impeding clinical translation. Therefore, optimizing the preparation process of complex‐structured, responsive RNA drug delivery nanocarrier to achieve consistent batch‐to‐batch quality and large‐scale manufacturing capacity is a critical issue to be addressed. In addition, the potential toxicity and systemic clearance of stimuli‐responsive RNA NPs also calls for in‐depth exploration. Other parameters, including extracellular and intracellular barriers as well as cell/tissue specificity and accumulation, should also be taken into account. It is noteworthy that the current main focus of stimuli‐responsive nanotechnology for RNA delivery is in the treatment of cancers. Other diseases such as inflammation, infection, and cardiovascular diseases, may also benefit from stimuli‐responsive RNA delivery due to the considerable difference between the affected tissues and normal tissues.

We expect that our review of stimuli‐responsive nanocarriers for RNA delivery could provide comprehensive information on basic design, functional mechanisms, and construction of nanocarrier‐based stimuli‐responsive RNA delivery systems, as well as inspire researchers to embrace future opportunities, challenges, and implementation in this field, which would help design next generation tailor‐made advanced RNA nanotherapeutics.

## Conflict of Interest

The authors declare no conflict of interest.
